# A New Antibiotic-Loaded Sol-Gel can Prevent Bacterial Intravenous Catheter-Related Infections

**DOI:** 10.3390/ma13132946

**Published:** 2020-07-01

**Authors:** John Jairo Aguilera-Correa, Rosa Vidal-Laso, Rafael Alfredo Carias-Cálix, Beatriz Toirac, Amaya García-Casas, Diego Velasco-Rodríguez, Pilar Llamas-Sillero, Antonia Jiménez-Morales, Jaime Esteban

**Affiliations:** 1 Clinical Microbiology Department, IIS-Fundacion Jimenez Diaz, UAM, 28040 Madrid, Spain; jestebanmoreno@gmail.com; 2 Hematology and Hemotherapy, IIS- Fundacion Jimenez Diaz, UAM, 28040 Madrid, Spain; rvidall@quironsalud.es (R.V.-L.); diego.velascor@quironsalud.es (D.V.-R.); pllamas@fjd.es (P.L.-S.); 3 Pathology Department, Fundacion Jimenez Diaz University Hospital, UAM, 28040 Madrid, Spain; rafael.carias@quironsalud.es; 4 Materials Science and Engineering Department, University Carlos III, 28911 Madrid, Spain; btoirac@ing.uc3m.es (B.T.); amayagarciacasas@gmail.com (A.G.-C.); toni@ing.uc3m.es (A.J.-M.); 5 Álvaro Alonso Barba Technological Institute of Chemistry and Materials, Carlos III University, 28911 Madrid, Spain

**Keywords:** catheter-related bloodstream infection, *Staphylococcus epidermidis*, anti-infective biomaterials, antibiotic-loaded sol-gel, local drug delivery of moxifloxacin, coagulation

## Abstract

The aim of this study was to evaluate the effectiveness of a moxifloxacin-loaded organic–inorganic sol-gel (A50) by locally preventing the catheter-related bloodstream infection (CRBSI) provoked by *Staphylococcus epidermidis* (*S. epidermidis*) and the effect resulting from its hydrolytic degradation on coagulation by using a rabbit in-vivo model. A50 coating can completely inhibit growth and would locally prevent CRBSI provoked by *S. epidermidis*. None of the coagulation blood parameters showed a significant difference constant over time between the control catheter group and the A50-coated catheter group, despite the visible silica release resulting from physiological A50 sol-gel degradation detected in serum at least during the first week. At pathological level, foreign body reaction was present in both of types of catheter, and it was characterized by the presence of macrophages and foreign body giant cell. However, this reaction was different in each group: the A50-coated catheter group showed a higher inflammation with histiocytes, which were forming granuloma-like aggregates with an amorphous crystalline material inside, accompanied by other inflammatory cells such as plasma cells, lymphocytes and mast cells. In conclusion, A50 coating a venous catheter showed excellent bactericidal anti-biofilm response since it completely inhibited *S. epidermidis* biofilm development and, far from showing procoagulant effects, showed slightly anticoagulant effects.

## 1. Introduction

More than half of hospitalized patients use a peripheral intravenous catheter (PIVC) and over one billion of these devices are used per year around world [1]. PIVCs are related to high rates of complications: for instance, insertion difficulty, phlebitis, infiltration, occlusion, dislodgment, and catheter-related bloodstream infection (CRBSI) [2]. CRBSI is defined as the presence of bacteremia originating from an intravenous catheter. This infection is one of the most frequent, lethal, and costly complications of central venous catheterization and also the most common cause of nosocomial bacteremia [3], which is associated with high mortality [4].

Classically, Gram-positive cocci—e.g., coagulase-negative staphylococci (CoNS) (mainly *Staphylococcus epidermidis*) (34.1%), enterococci (16%), and *Staphylococcus aureus*, 9.9%—are the most common, followed by *Candida* species (11.8%), and Gram-negative bacilli—*Klebsiella*, 5.8%; *Enterobacter*, 3.9%; *Pseudomonas*, 3.1%; *Escherichia coli*, 2.7%; *Acinetobacter*, 2.2%—and other bacteria (10.5%) [5]. However, the incidence of CoNS, *S. aureus* and yeasts decreased significantly over time, while Gram-negative bacilli and enterococci remained stable over time in terms of CRSBSI per 1000 admissions [6]. For all these organisms, biofilm development on the surface of indwelling catheters is central to the pathogenesis of this infection [7].

CRBSI-preventive strategies can be included the following: to use maximal sterile barriers for insertion and chlorhexidine for skin antisepsis, to avoid lower-extremity insertion sites, to remove the catheter when no longer needed, to promote good hand hygiene, to designate an intravenous therapy team, and to use antimicrobial-impregnated catheters [7]. Antimicrobial catheters are impregnated in a single antibiotic, like vancomycin or cefazolin, or a couple of antibiotics, for instance, chlorhexidine–silver sulfadiazine, rifampin–minocycline, or rifampin–miconazole [8,9]. Antimicrobial catheter strategies which go beyond the impregnation method are less common; however, it is worth emphasizing catheters made of polyurethane supersaturated with molecularly dispersed rifampin and miconazole [10]. Recently, we demonstrated that a moxifloxacin-loaded organic–inorganic sol-gel is able to locally prevent the prosthetic joint infection provoked by both Gram-positive and Gram-negative bacteria [11]. This antibacterial activity is a result of the hydrolytic degradation of organic–inorganic sol-gel [12], which favors a lineal release of moxifloxacin from the material [11]. Sol-gel technology is a versatile tool, with an easy, environmentally friendly and low-energy-consuming procedure, which, unlike other synthesis processes to obtain organic polymer coatings [13,14], does not involve performing several procedural steps nor pretreatment of the biomaterial surface [12,15].

The aim of this study was to evaluate the effectiveness of a moxifloxacin-loaded organic–inorganic sol-gel by preventing locally the CRBSI caused by *Staphylococcus epidermidis* and the effect resulting from its hydrolytic degradation on coagulation by using a rabbit in-vivo model.

## 2. Material and Methods

### 2.1. Sol-Gel Synthesis and Catheter Coating

Sol-gel was prepared as described elsewhere [16], starting with a mixture of *γ*-methacryloxypropyltrimethoxysilane 98% (Acros Organics, Thermo Fisher Scientific, Waltham, MA, USA) and tetramethylorthosilane 98% (Acros Organics, Thermo Fisher Scientific, Waltham, MA, USA) with a molar ratio of 1:2. Later, tris (trimethylsilyl) phosphite 92% (Sigma Aldrich, Saint Louis, MO, USA) was added at a molar ratio of 1:52 with regard to silanes. Moxifloxacin (Sigma Aldrich, Saint Louis, MO, USA) dissolved in water was added at a concentration of 50 mg per 20.3 mL of sol-gel (A50). This concentration has been intentionally chosen because it represents the maximum amount of antibiotic which sol-gel can contain without compromising its stability, durability and adherence to titanium substrates [11]. The physicochemical characterization of sol-gel has already been previously described [12]. The moxifloxacin release rate from a moxifloxacin sol-gel under physiological conditions was 120 ng/h [11].

**Figure 1:**
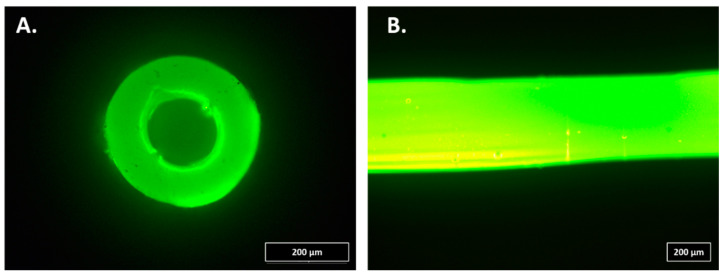
Fluorescence micrographs of a catheter intraluminally (**A**) or extraluminally (**B**) coated with A50 sol-gel. The fluorescence detected is uniquely due to the presence of moxifloxacin trapped by the net of the sol-gel.

For the infection study, 20-cm soft Premicath (Vygon, Swindon, UK) was intraluminally coated with A50 sol-gel. For this purpose, 200 µL of A50 sol-gel was injected into the catheters. Then, each catheter was connected to a sterile airflow pumped by a single-channel peristaltic pump (Watson-Marlow 101U/R MK2, Watson-Marlow, Barcelona, Spain) for 24 h at room temperature in a laminar flow cabinet (Figure 1A).

For the coagulation study, 20-cm soft Premicath was extraluminally coated with A50 sol-gel. For this purpose, each catheter was coated with A50 sol-gel by a spray method, and, then, it was vertically hung and dried for 24 h at room temperature in a laminar flow cabinet (Figure 1B).

### 2.2. In-Vitro Study

The *S. epidermidis* ATCC35984 strain was used to assess the bactericidal of sol-gel. The strain was kept frozen at −80 °C until experiments were performed. The strain was grown overnight in a blood tryptic-soy agar plate at 37 °C and 5% CO_2_. A MBEC^TM^ biofilm incubator lid (Innovotech, Edmonton, AB T6N 1H1, Canada) was coated by dipping in wells filled with 200 μL of non-loaded sol-gel and moxifloxacin-loaded sol-gel (A50).Lids were then dried for at least 20 h at room temperature in a laminar flow cabinet. Uncoated pegs were used as a positive control. This experiment was performed in triplicate and 8 wells per replicate (24 well per condition). An inoculum of the strain and tryptic-soy broth (TSB) supplemented with 1% of glucose [17] was adjusted to 1.6 × 10^6^ colony-forming units per mL, favoring the *S. epidermidis* biofilm formation. Two hundred microliters of the bacterial inoculum were deposited on each well of a 96-well flat-bottom plate. The 96-well plate was closed with the lid coated by the sol-gels and the biofilm was grown for 24 h at 37 °C and 5% CO_2_. The biofilm of each well was rinsed two times with 200 μL of saline with 0.9% NaCl (B.Braun, Melsungen, Germany). Two hundred microliters of TSB with 10% of alamarBlue^®^ (BIO-RAD, Hercules, CA, USA) [18] were added to each well, and the plate was incubated for 30 min at 80 rpm at 37 °C. Fluorescence was measured using an excitation and emission wavelength of 560 nm and 590 nm, respectively, by using an EnSpire Multimode Plate Reader (Perkin Elmer, Waltham, MA, USA).

### 2.3. Infection Model

We used a clinical strain of *Staphylococcus epidermidis* from a 67-year-old woman with a catheter-associated infection (Se) isolated in the Clinical Microbiology Department of the Fundación Jiménez Díaz University Hospital.

The Instituto de Investigación Sanitaria-Fundación Jiménez Díaz (IIS-FJD) Research Commission did not require the study to be reviewed or approved by an ethics committee because no clinical, demographical, analytical or any other data from the patient were included, and bacterial strains do not need such approval according to the present legislation.

This study was approved by the IIS-FJD Animal Care and Use Committee, which includes ad-hoc members for ethical issues. Animal care and maintenance complied with institutional guidelines as defined in national and international laws and policies (Spanish Royal Decree 53/2013, authorization reference PROEX174/17 21 November 2017 granted by the Counsel for the Environment, Local Administration and Territorial Planning of the Community of Madrid and, Directive 2010/63/EU of the European Parliament and of the Council of 22 September 2010).

Specific pathogen-free New Zealand white male rabbits (Granja San Bernardo, Navarra, Spain) of between 3.5 and 4.5 kg in weight were used. All animals were individually housing in individual cages in an air-conditioned room at 22 ± 2 °C and cycles of light–darkness of 12:12 h.

Prior to catheter insertion, each catheter was infected by injecting 200 µL of brain–heart broth supplemented with 2% glucose and 10^6^ colony-forming units (CFU) per mL (CFU/mL) of *S. epidermidis*. After injection, each catheter was incubated at 37 °C and 5% CO_2_ for 24 h. After 24-h incubation, each catheter was rinsed with 200 µL of sterile saline (B. Braun, Melsungen, Germany).

**Figure 2:**
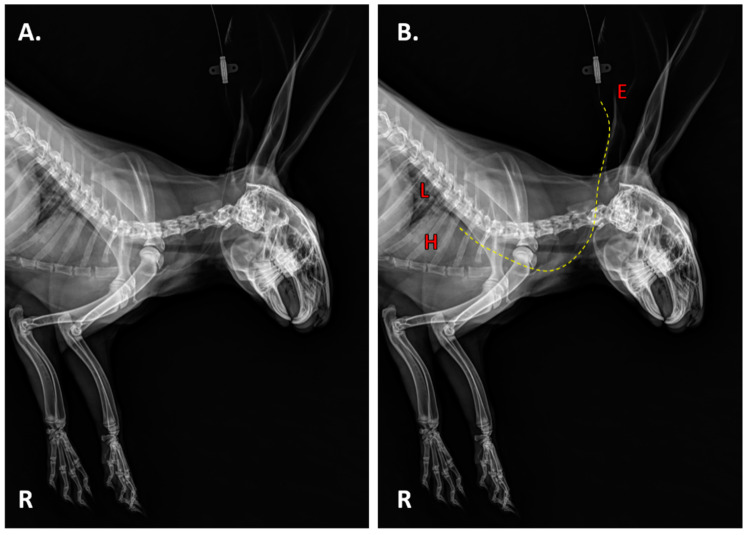
Lateral radiography of a rabbit with a catheter in its marginal ear veins (**A**) and its anatomical location (**B**). The discontinuous yellow line represents the catheter path. E: ear. L: lung. H: heart.

Sixteen centimeters of an A50-coated or uncoated infected Premicath per ear (two catheters per rabbit) was aseptically inserted into the marginal ear vein of rabbits (Figure 2); they were anesthetized and 200 µL of blood was extracted using a sterile 1-mL syringe and injected into a pediatric blood-culture bottle and incubated in BacT/ALERT^®^ 3D (Biomérieux, Marcy-l’Étoile, France) for at maximum of 120 h. Each catheter was fixed to the ear by a simple point using a PRONOVA 5/0 suture and cut to skin level. Three rabbits carried two uncoated catheters (*n* = 6) and three other rabbits carried two A50-coated catheters (*n* = 6).

After insertion, blood samples were periodically and aseptically extracted from each animal under general anesthesia for hematological analysis. Furthermore, the weight and temperature of each rabbit was measured every 24 h. Three weeks after the catheter insertion, each animal was euthanized under general anesthesia by an intracardiac overdose of sodium thiobarbital. The two catheters were recovered through sterile preparation of each ear.

Each catheter was aseptically cut up in 1-cm pieces; all these pieces were immersed in 10 mL of sterile saline and sonicated using an Ultrasons-H 3,000,840 low-power bath sonicator (J. P. Selecta, Barcelona, Spain) at 22 °C for 5 min. The resulting sonicate was diluted in a 10-fold dilution bank, seeded on blood–chocolate agar (Biomérieux, Marcy-l’Étoile, France) using the spread plate method, and incubated at 37 °C and 5% CO_2_ for 24 h. The concentration of bacteria was estimated as CFU/cm^2^ of catheter.

The first centimeter of some catheters of each experimental group was examined using scanning electron microscopy. For this, catheter pieces were fixed on the alloys with 2.5% glutaraldehyde in 0.1 M sodium cacodylate buffer at pH 7 at 4 °C for 45 min. Samples were then dehydrated with increasing concentrations of ethanol (30%, 50%, 70%, 90% and 100%) at 22 °C for 10 min. Micrographs were obtained using a Teneo FEI tungsten filament electronic microscope (Field Electron and Ion Company, FEI, OR, USA).

### 2.4. Coagulation Study

For this study, 16 cm of an A50-coated or uncoated infected Premicath per rabbit (one catheter per rabbit) was aseptically inserted into the marginal ear vein of rabbits anesthetized and 200 µL of blood was extracted by using a sterile 1-mL syringe. Each catheter was fixed to the ear by a simple point by using a PRONOVA 5/0 suture and cut to skin level. Three rabbits carried two uncoated catheters (*n* = 3), and three other rabbits carried two A50-coated catheters (*n* = 3).

After insertion, blood samples were periodically and aseptically extracted from each animal under general anesthesia for silica blood concentration and hematological and coagulation analysis. Hematological analysis was performed by the Hematology Clinical Analysis Department of Fundación Jiménez Díaz University Hospital.

For the basic coagulation test, whole blood samples were collected in a sodium citrate containing tube (nine parts blood to one part 0.105 M sodium citrate) and subsequently centrifuged at 3000× *g* for 15 min at room temperature. For thrombin generation assay, a double centrifugation was performed, with plasma decanted, aliquoted and stored at −80°C until testing.

Fibrinogen was determined by using a Fibrinogen Clauss assay calibrated with a HemosIL Calibration Plasma kit (Instrumentation Laboratories, Bedford, MA, USA), which contains a standard reference material specific to this particular coagulation analyzer and its reagent. Prothrombin time (PT) was determined on an ACL Top 700 analyzer (Instrumentation Laboratory, Bedford, MA, USA). The silica concentration in serum over time was estimated by atomic absorption spectroscopy by using a Perkin Elmer Analyst 600 in Reference Laboratories (Barcelona, Spain).

For the assessment of thrombin generation, calibrated automated thrombin generation (TG) was measured in platelet-poor plasma using the ST Genesia^®^ analyzer (Diagnostica Stago, Asnières sur Seine, Cedex, France). The ST Genesia^®^ is a fully-automated system for measuring TG according to the reference CAT assay of Hemker et al. [19]. The calibration was done with a mixture of thrombin solution (STG^®^-ThrombiCal, Stago, Asnières-sur-Seine, France) and a combination of fluorogenic substrate (amino-methylcoumarin, AMC) and calcium chloride (STG^®^-FluoStart, Stago, Asnières-sur-Seine, France). This calibration curve was then scaled to each plasma sample through the measurement of a fixed quantity of the fluorophore by time, in parallel to both the calibration and rabbit plasma mixture to convert the fluorescence to quantity of thrombin. The calibration fluorescence over time also produced a straight line after correction of the inner filter effect and fluorogenic substrate consumption along reaction. STG^®^-FluoSet was used as a measure of absorption characteristics (colour, turbidity, etc.) for each sample to compensate for them. The TG in rabbit plasma samples was measured using 80 μL of plasma with 2.5 pM of human recombinant tissue factor (TF) and 2 µM of synthetic phospholipids in a 1:2 dilution of PPP in buffer solution Thrombinoscope (BV, Stago, Asnières-sur-Seine, France). TG was then monitored after the addition of a fluorogenic thrombin substrate and calcium chloride (STG-FluoStart). Therefore, the final test mixture was composed of 80 μL of plasma, 20 μL of trigger (TF and phospholipids) and 20 μL of STG-FluoStart solution. The thrombin produced in the rabbit plasma samples converted the substrate into AMC, which generates a fluorescent signal. The increasing fluorescence signal was regularly measured and followed in time. The instrument combines the fluorescence generated on the sample cuvette over time with the calibration and the absorption characteristics information to make all the corrections and to get the kinetics of thrombin quantity generated by time in each sample. The following TG-derived parameters were assessed in all rabbits: lag time (number of minutes needed to start the thrombin formation), peak height (maximum amount of thrombin produced at any time), time to peak (number of minutes needed to reach the highest amount of thrombin), and ETP (area under the curve, total amount of thrombin produced).

Four weeks after catheter insertion, each animal was euthanized under general anesthesia by an intracardiac overdose of sodium thiobarbital. The catheter was recovered through sterile preparation of the ear, and at least 5 cm of this ear was cut for pathological study. For the pathological study, marginal ear veins were fixed, paraffin-infiltrated, cut in transversal sections and stained with hematoxylin–eosin.

The first centimeter of some catheters of each experimental group was examined using scanning electron microscopy following the above-mentioned methodology (see Section 2.3. *Infection model*).

### 2.5. Statistical

Statistical analyses were performed using Stata Statistical Software, Release 11 (StataCorp 2009, College Station, TX, USA). Data were evaluated using the one-sided pairwise Wilcoxon nonparametric test, considering significant a level of statistical significance of *p* ≤ 0.05. All data are represented as median and interquartile using GraphPad PRISM 6.01 (GraphPad Software 2012, San Diego, CA, USA).

## 3. Results

### 3.1. In-Vitro Study

**Figure 3:**
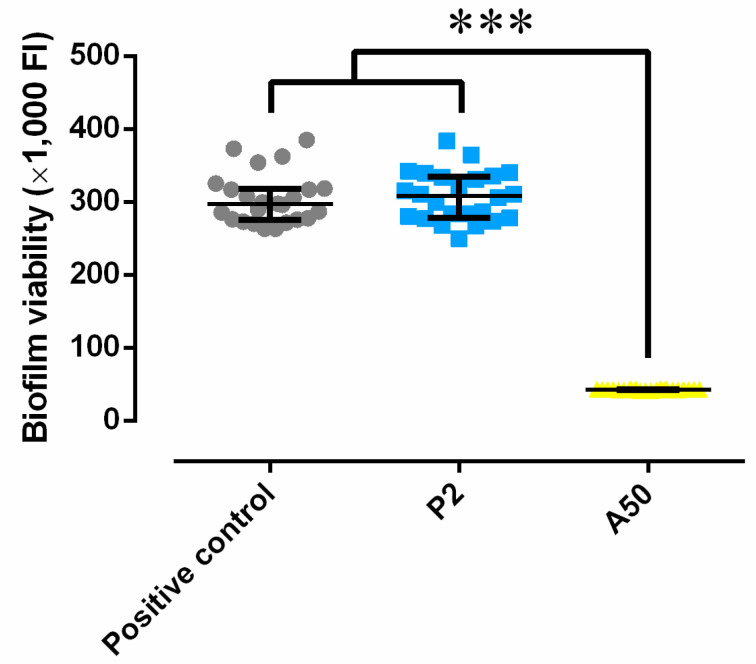
Biofilm viability of *S. epidermidis* ATCC35984 developed in the presence of sol-gel without antibiotic (P2) and of moxifloxacin-loaded sol-gel (A50). ***: *p*-value < 0.001 for Wilcoxon test between the groups compared.

The antibacterial effectiveness of the coatings was analyzed through the viability of the biofilm grown in the presence of each type of sol-gel, and the results obtained are represented in Figure 3. The results revealed that A50 completely and significantly inhibited the *S. epidermidis* ATCC35984 biofilm development (*p*-value < 0.0001) relative to the positive control and non-loaded sol-gel (P2).

### 3.2. In-Vivo Model

#### 3.2.1. Infection Model

The median and the interquartile range of blood culture time to positivity of rabbit blood extracted after catheter insertion from control catheter group was significantly higher than A50-coated catheter group (*p*-value = 0.0009): 1 (1–1) h for control catheter group and 15.5 (15.5–54.5) h.

**Figure 4:**
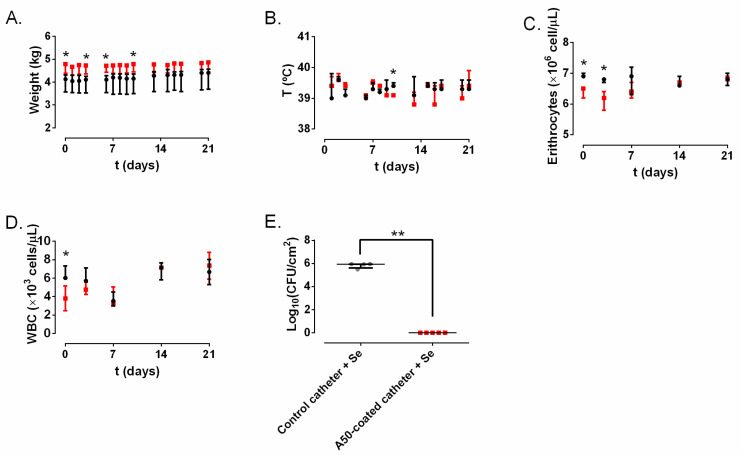
*S. epidermidis* (Se) infection model results of the control catheter group (black) and A50-coated catheter group (red) over time: weight (**A**); rectal temperature (**B**); erythrocytes (**C**); white blood cells (WBC) (**D**); and the quantity of bacteria per intraluminal area unit (**E**). *: *p*-value < 0.05, **: *p*-value < 0.01 for Wilcoxon test between the control catheter group and A50-coated catheter group.

Three catheters were removed by the rabbits which brought them: two from the control group and one from the A50-coated catheter group throughout the study. The weight of the rabbit group with *S. epidermidis* (Se)-infected A50-coated catheters was slightly higher than the control groups (Figure 4A). Non-statistically-significant differences in temperature were observed over time between both groups (Figure 4B). The most important hematological results related to infection are shown in Figure 4C,D: erythrocytes and white blood cells show a slight but statistically significant difference between groups at day 0.

The amount of bacterial concentration per area unit in the inner of the Se-infected control catheter group was significantly higher than in the Se-infected A50-coated catheter group (*p*-value = 0.0037) (Figure 4E).

**Figure 5:**
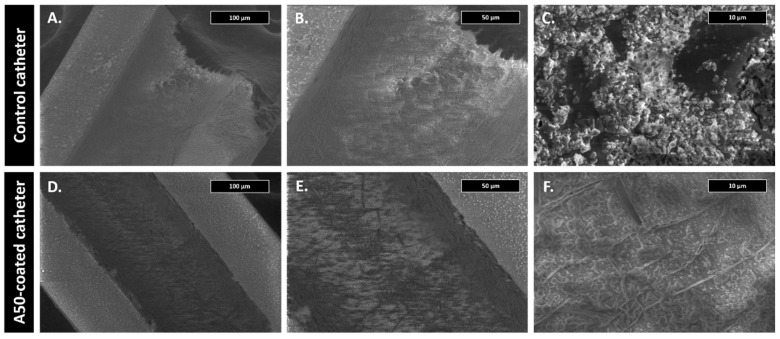
Scanning electron microscopy micrographs of each type of catheter after 21 days in the marginal ear vein from the infection model at 300× (**A**,**D**), 600× (**B**,**E**), and 5000× magnifications (**C**,**F**).

Well-formed staphylococcal biofilms and cocci with blood cell debris were observed in the internal lumen of control catheters. This staphylococcal biofilm was not present in the A50-coated catheters, where only isolated cocci and blood cells debris adhered to A50 sol-gel could be observed (Figure 5).

#### 3.2.2. Coagulation Model

**Figure 6:**
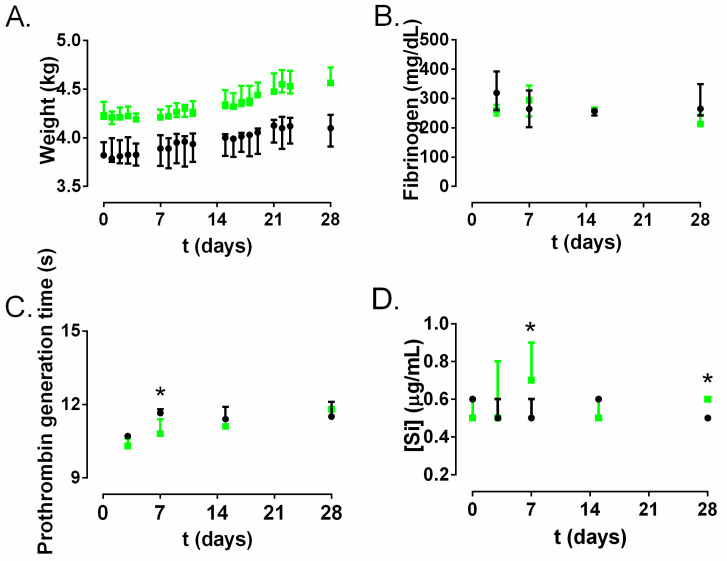
Hematological coagulation model results of control catheter group (black) and A50-coated catheter group (green) over time: weight (**A**); fibrinogen (**B**); prothrombin generation time (**C**); and silica concentration in serum (**D**). *: *p*-value < 0.05 for Wilcoxon test between control catheter group and A50-coated catheter group.

The weight was significantly different between both of the rabbit groups studied (Figure 6A). Non-significant differences were found in the fibrinogen (Figure 6B) and prothrombin generation times (Figure 6C). The silica concentration in the serum over time in the A50-coated catheter group showed a slight but growing trend which reached the maximum concentration at day 7, where it was significantly higher than the control catheter group (*p*-value = 0.0248); from this day, this difference disappears (Figure 6D).

**Figure 7:**
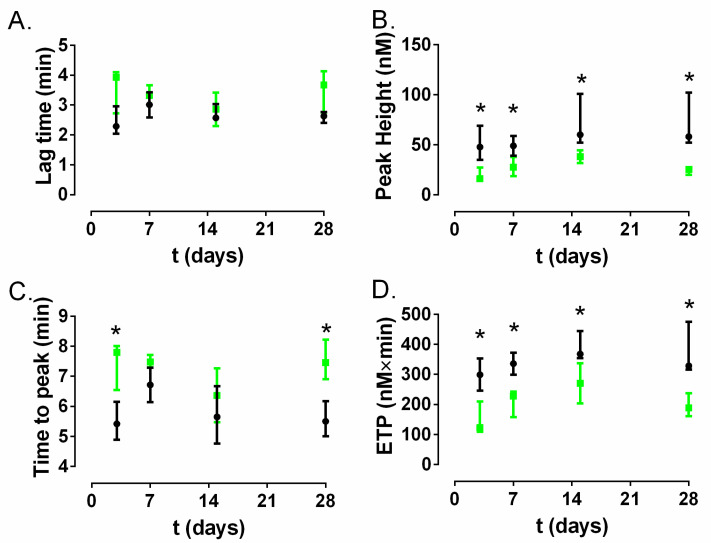
Coagulation model results obtained with ST Genesia^®^ for the control catheter group (black) and the A50-coated catheter group (green) over time: time to start thrombin generation (lag time) (**A**); time to reach the maximum peak of thrombin (time to peak) (**B**); the maximum peak of thrombin formation (peak height) (**C**) and the total amount of thrombin generated (endogenous thrombin potential, ETP) (**D**). *: *p*-value < 0.05 for Wilcoxon test between the control catheter group and A50-coated catheter group.

The results obtained with ST Genesia^®^ are shown in Figure 7 Non-significant differences were found in the time to start thrombin generation (lag time) (Figure 7A). The time to reach the maximum peak of thrombin (time to peak) was significantly lower in the A50-coated catheter group than in the control catheter group at days 3 and 28 (*p*-value = 0.0248) (Figure 7B). The maximum peak of thrombin formation (peak height) was significantly reduced in the A50-coated catheter group compared to the control catheter group at days 3, 14 and 28 (*p*-value = 0.0248) (Figure 7C). The total amount of thrombin generated (endogenous thrombin potential, ETP) was significantly lower in the A50-coated catheter group than in the control catheter group at days 3, 7, 14 and 28 (*p*-value = 0.0248) (Figure 7D).

**Figure 8:**
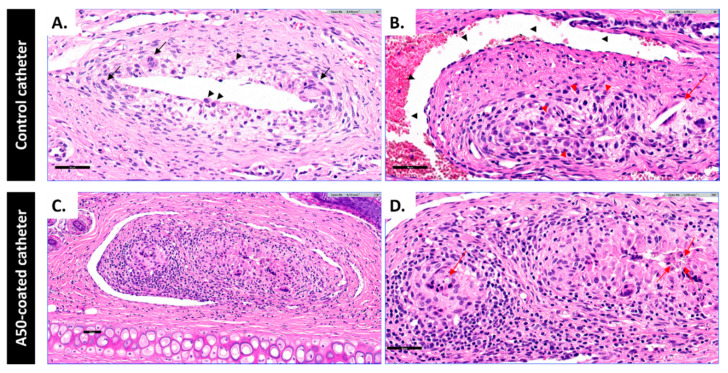
Representative images stained with hematoxylin–eosin of rabbit marginal ear veins from each group from the coagulation model: (**A**) granulomatous inflammation with histiocytes (black arrows) which were not forming granuloma-like aggregates; (**B**) inflammatory pseudothrombotic injury lined by a layer of endothelial cells (black arrows) with giant cells (red arrows); (**C**) well-formed granulomas with plasma cells, lymphocytes and scarce mast cells; and (**D**) granuloma with multinucleated giant cells contained an amorphous crystalline material inside (red arrows). The bar represents 50 µm.

At the pathological level, both the control catheter group and A50-coated catheter group showed a chronic granulomatous inflammation conformed by histiocytic cells (Figure 8A, black arrows), accompanied or not by granuloma formation with giant cells, a typical aspect of a foreign body reaction. However, there were differences between the control catheter group and A50-coated catheter group. The control catheter group showed a granulomatous inflammation with histiocytes (Figure 8A, black arrows) which were not forming granuloma-like aggregates. It is noteworthy that one of the three marginal ear veins showed an inflammatory pseudothrombotic injury lined by a layer of endothelial cells (Figure 8B, black arrows) with giant cells (Figure 8B, red arrows). For its part, the A50-coated catheter group showed a granulomatous inflammation with histiocytes with well-formed granulomas with plasma cells, lymphocytes and scarce mast cells (Figure 8C). Furthermore, these granulomas with multinucleated giant cells contained an amorphous crystalline material inside (Figure 8D, red arrows).

**Figure 9:**
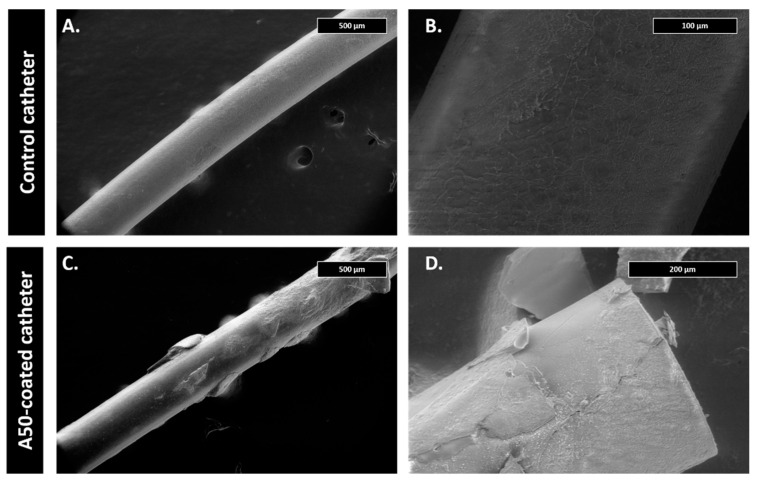
Scanning electron microscopy micrographs of each type of catheter after 28 days in the marginal ear vein from the coagulation model at 50× (**A**,**C**), and 200× magnifications (**B**,**D**).

At the microscopic level, the control catheter and A50-coated catheter were coated by the typical fibrous capsule of foreign body reaction; however, the A50-coated catheter showed small areas where A50 sol-gel still remained after 28 days (Figure 9).

## 4. Discussion

In this study, we report the efficacy of a novel approach to moxifloxacin-loaded sol-gel coatings, which was demonstrated to provide an anti-infective surface associated with intravenous catheters. The active surfaces locally prevented biofilm development in vitro and also biofilm development in vivo of *Staphylococcus epidermidis*, a clinically important bacterial species. Finally, we demonstrated the in-vivo anticoagulation effect of moxifloxacin-loaded sol-gel.

The infection model showed that A50-coated catheters start to prevent *S. epidermidis* infection both in in-vitro and in-vivo studies. It is noteworthy that, in the in-vivo model, bacterial biofilm development was prevented even before insertion into marginal ear veins, since the blood culture time to positivity was significantly higher than with the control catheters (*p*-value < 0.001), although the growth medium (brain–heart infusion + 2% glucose) favored bacterial growth and biofilm development [17]. The blood culture time to positivity is an indirect measure of initial bacterial inoculum and/or growth rate. Thus, the lower the time to positivity, the higher the inoculum in the blood and/or the higher the growth rate [20]. Rabbits did not show signs of systemic infection: they kept on gaining weight during all the experiments, and none of them had fever, or hematological alterations (Figure 4A–D). In cases of septicemia and in bacterial infections, leukopenia and reduced total red blood cells used to be present [21]. In our case, these values did not show any alteration or significantly constant difference over time between both groups; therefore, it can be said that the infection remained confined in the lumen of the catheter and did not provoke a CRBSI. Our results show that A50 coating can completely inhibit the growth and would locally prevent CRBSI provoked by *S. epidermidis* (Figure 4E), the most common pathogen related to this kind of infection [22], but it would also inhibit other bacteria related to this infection, such as other CoN staphylococci, some enterobacteria, and even *S. aureus* [22]. These findings are consistent with a previous in-vivo report, which revealed that A50-coated titanium implants can completely prevent prosthetic joint infection caused by *S. aureus* and *Escherichia coli* [11]. This infection model is not exempt from at least three limitations. Firstly, *S. epidermidis* catheter-associated infection is extraluminal and not intraluminal [23]. However, *S. epidermidis* intraluminal infection had to be carried out, since its extraluminal infection can easily be cleared by the immune system from rabbits [24]. Secondly, comparing the start inoculum in both groups of infected catheters, the A50-coated catheter inhibited bacterial growth and biofilm development before insertion. Nevertheless, our results bring out that whatever the initial inoculum of bacteria, no catheter-associated infection developed on A50-coated catheters. Thirdly, though it is true that bacterial infections cause leukopenia in rabbits, its hemogram usually shows a shift from lymphocyte-predominant to neutrophil-predominant differential counts [21]. This shift cannot be detected with the instruments used by the Clinical Analysis Department of our hospital.

The antibacterial activity of moxifloxacin-loaded sol-gel is a fruit of moxifloxacin release resulting from hydrolytic degradation of organic–inorganic sol-gel [11] during its hydration in contact with systemic fluids [12]. Sol-gel degradation in physiologic conditions gives rise to different debris and/or silica-derivate molecular components from sol-gel, which may interact with the blood, causing coagulation complications, or with the walls of the veins with which the catheter is in contact. Fibrinogen and prothrombin are liver-generated proteins which play roles in blood clotting and the inflammatory response, among other things [25,26]. Abnormal values of these parameters can be related to abnormalities in blood coagulation or inflammatory processes [26,27]. According to our results, none of them showed a significant difference constant over time between the control catheter group and A50-coated catheter group (Figure 5B,C) despite the visible silica release from A50 sol-gel detected in serum at least during the first week (Figure 5D). These findings indicate that there were no differences in these coagulation parameters between the control catheter and A50-coated catheter group. Furthermore, the coagulation state can be also inferred from analysis of the thrombin generation assay [27], where the ETP and peak values indicated a significantly lower state of hypercoagulability over time for the A50-coated catheter group compared to the control catheter group (*p*-value < 0.05), although the lag time and the time to peak almost did not show significant differences. Our findings may be cosidered contrary to the findings reported by other authors who asserted that silica nanoparticles can trigger the systemic activation of coagulation cascade and platelets in in-vivo models [28,29,30].

The inflammatory potential of the A50-sol-gel was explored by evaluating the risk of vein inflammation associated with inserting A50-coated catheters. Host reactions following implantation of a catheter include injury, blood–catheter interactions, provisional matrix formation, acute inflammation, chronic inflammation, granulation tissue development, foreign body reaction, and fibrous capsule development [31]. This foreign body reaction was present in both of types of catheter, and it was characterized by the presence of macrophages and foreign body giant cells [31]. However, this reaction was different in each group: the A50-coated catheter group showed a higher inflammation with histiocytes, which were forming granuloma-like aggregates with an amorphous crystalline material inside, accompanied by other inflammatory cells such as plasma cells, lymphocytes and mast cells. The amorphous crystalline material may be the debris of A50 sol-gel resulting from sol-gel hydrolytic degradation [12]. The presence of sol-gel debris may prompt the recruitment of macrophages on the implantation site and impede the bacterial proliferation according to a previous work [11]. It is worth highlighting that the presence of mast cells in the A50-coated catheter group might explain the systemic anticoagulant effect of sol-gel, since mast cells produce heparin and another anionic polysaccharide, chondroitin sulphate [32], both of them with important effects on coagulation cascade [33,34]. This in-situ pathological response provoked by the sol-gel may explain the similarity of A50-coated and heparin-coated catheters [35], since both of them presented a higher inflammation rate than their respective controls.

## 5. Conclusions

Moxifloxacin-loaded sol-gel coating of a venous catheter showed an excellent bactericidal anti-biofilm response since it completely inhibited *S. epidermidis* biofilm development and, far from showing procoagulant effects, showed slightly anticoagulant effects.

## 6. Patent

The sol-gel used in this study is one of the possible products protected by the Spanish patent with Publication Number 2686890, applied for 19th April 2017, and entitled *Procedure for Obtaining a Sol-Gel Coating, Composition Coating and Use of the Same*. All authors of this patent have contributed to this work.
